# Epistatic Interaction of *ERAP1* and HLA-B in Behçet Disease: A Replication Study in the Spanish Population

**DOI:** 10.1371/journal.pone.0102100

**Published:** 2014-07-14

**Authors:** Marta Conde-Jaldón, Marco Antonio Montes-Cano, José Raul García-Lozano, Lourdes Ortiz-Fernández, Norberto Ortego-Centeno, Rocío González-León, Gerard Espinosa, Genaro Graña-Gil, Juan Sánchez-Bursón, Miguel Angel González-Gay, Ana Celia Barnosi-Marín, Roser Solans, Patricia Fanlo, Mónica Rodríguez Carballeira, Teresa Camps, Santos Castañeda, Javier Martín, María Francisca González-Escribano

**Affiliations:** 1 Servicio de Inmunología, IBiS, H.U. Virgen del Rocío/CSIC/Universidad de Sevilla, Sevilla, Spain; 2 Servicio de Medicina Interna, H. Clínico San Cecilio, Granada, Spain; 3 Servicio de Medicina Interna, H. U. Virgen del Rocío, Sevilla, Spain; 4 Servicio de Enfermedades Autoinmunes, H. Clinic, Barcelona, Spain; 5 Servicio de Reumatología, CHU A Coruña, Spain; 6 Servicio de Reumatología, H. de Valme, Sevilla, Spain; 7 Servicio de Reumatología, H. Marques de Valdecilla, Santander, Spain; 8 Servicio de Medicina Interna, H. Torrecárdenas, Almería, Spain; 9 Servicio de Medicina Interna, H. Vall d'Hebron, Barcelona, Spain; 10 Servicio de Medicina Interna, H. Virgen del Camino, Pamplona, Spain; 11 Medicina Interna Unitat de Malalties Autoimmunes i Sistèmiques Hospital Universitari Mútua Terrassa, Barcelona, Spain; 12 Servicio de Medicina Interna, H. Carlos Haya, Málaga, Spain; 13 Servicio de Reumatología, H. de la Princesa, Madrid, Spain; 14 Instituto de Parasitología y Biomedicina López Neyra, CSIC, Granada, Spain; Albert Einstein Institute for Research and Education, Brazil

## Abstract

Behçet's disease (BD) is a multifactorial disorder associated with the HLA region. Recently, the ERAP1 gene has been proposed as a susceptibility locus with a recessive model and with epistatic interaction with HLA-B51. ERAP1 trims peptides in the endoplasmic reticulum to optimize their length for MHC-I binding. Polymorphisms in this gene have been related with the susceptibility to other immune-mediated diseases associated to HLA class I. Our aim was, the replication in the Spanish population of the association described in the Turkish population between ERAP1 (rs17482078) and BD. Additionally, in order to improve the understanding of this association we analyzed four additional SNPs (rs27044, rs10050860, rs30187 and rs2287987) associated with other diseases related to HLA class I and the haplotype blocks in this gene region. According to our results, frequencies of the homozygous genotypes for the minor alleles of all the SNPs were increased among patients and the OR values were higher in the subgroup of patients with the HLA-B risk factors, although differences were not statistically significant. Moreover, the presence of the same mutation in both chromosomes increased the OR values from 4.51 to 10.72 in individuals carrying the HLA-B risk factors. Therefore, although they were not statistically significant, our data were consistent with an association between *ERAP1* and BD as well as with an epistatic interaction between *ERAP1* and HLA-B in the Spanish population.

## Introduction

Behçet disease (BD) is a multifactorial disorder with evidence of genetic contribution based on familial aggregation, predominance in patients with Mediterranean or Asian ancestry and association with HLA-B51 in several ethnic groups [1]–[4]. Contribution of the HLA region has been estimated to represent approximately 20% of the genetic component of this disease [5]. In addition to the HLA class I region, in which different peaks of association have been detected, a relationship of the disease with IL23R and IL10 has been described using a large scale SNP genotyping strategy (genome wide association studies, GWAS) in different Caucasian and Asian populations [6]–[11].Very recently, the *ERAP1* gene has been proposed as a new susceptibility locus for BD in a Turkish patient cohort [12].

The *ERAP1* gene is located on chromosome 5q15 and it encodes an amino-peptidase with an ubiquitous tissue distribution. This enzyme is responsible for peptides N-terminal trimming in the endoplasmic reticulum which is a critical step of the processing of the peptides to optimize their length for MHC-I binding [13], [14]. Moreover, ERAP1 has been involved in the cleaving of several proinflammatory cytokine receptors from the cell membrane including shedding of tumor necrosis factor receptor (TNFR1), IL-1 receptor-II (IL-RII) and IL-6 receptor α (IL-6 α) [15]–[17] although this function remains to be confirmed. Therefore, from a functional perspective, *ERAP1* represents an excellent biological candidate for association with BD. In fact, this gene has been related with the susceptibility to other immune-mediated diseases associated to HLA class I such as ankylosing spondylitis (AS) and psoriasis [18]–[21] which have also been associated with IL-23R and IL10 suggesting the existence of shared pathogenesis pathways [9]–[11], [18], [22], [23]. In their study, Kirino et al [12], described a recessive model of association with BD in a Turkish population and they found an epistatic interaction between HLA-B51 and *ERAP1* which could not be replicated in a Japanese cohort [12]. The objective of the present study was to asses the association of *ERAP1* with BD in the Spanish population.

## Methods

A total of 362 BD patients (148 males and 214 females) with a mean age at onset ± SD of 35.1±11.2 fulfilled the 1990 International Study Group classification criteria for Behçet's disease [24] and 460 bone marrow and blood donors as normal healthy controls (50% males) were included in the study. All the subjects were Spanish European recruited from different Spanish hospitals. The study was approved by all local ethical committees of the corresponding hospitals A Coruna (CHU A Coruña), Almería (H. Torrecárdenas), Barcelona (H. Clinic, Vall d'Hebron and Mútua Terrassa), Granada (H. Clínico San Cecilio), Madrid (H. de la Princesa), Málaga (H. Carlos Haya), Pamplona (H. Virgen del Camino), Santander; (H Marques de Valdecilla) and Sevilla (Virgen del Rocío and Valme). All the study participants gave written informed consent according to the declaration of Helsinki. Clinical features of the patient group were: 100% had oral ulcers, 75% genital ulcers, 50% uveitis, 50% arthritis, and 25% vascular, 18% neurological and 19% gastrointestinal involvement. The distribution of the frequencies of the different markers in the cohorts from different hospitals was not significantly different.

Peripheral blood was obtained from the healthy controls whereas peripheral blood or saliva was the starting material obtained from patients. Genomic DNA was extracted using QIAmp DNA Mini Kit (Qiagen, Barcelona, Spain) according to the manufacturer's recommendations and stored at −20°C. The purity of DNA was determined using a Nanodrop spectrophotometer (Thermo Fisher Scientific, Wilmington, DE 19810 U.S.A.). Only DNA samples having a 260/280 ratio between 1.7 and 2.0 and a final concentration of 10–20 ng/µl were considered appropriate. Ten DNA samples from saliva were eliminated because they did not meet these quality criteria.

Five non-synonymous single nucleotide polymorphisms (SNPs) associated with AS rs27044 (Gln730Glu), rs17482078 (Arg725Gln), rs10050860 (Asp575Asn), rs30187 (Arg528Lys) and rs2287987 (Val349Met) were included in this study. Genotyping of these SNPs was performed using TaqMan SNP Genotyping Assays (Applied Byosistems, Barcelona, Spain) in a LightCycler 480 (Roche, Barcelona, Spain). Patients and controls had been previously genotyped in HLA-A and HLA-B using PCR-SSOP Luminex method with LABType SSO (One Lambda Inc., Canoga Park, CA), following the manufacturer's instructions. According to our previous results HLA-B*51 and HLA-B*57 were considered as HLA-B risk factors in our population [25].

The *a priori* statistical power was calculated using the minor allele frequencies (MAF) described in the European population for the five SNPs included in this study (http://www.broadinstitute.org/mammals/haploreg/haploreg.php), taking into consideration a recessive association model of the minor alleles and the Odd Ratio (OR) value described by Kirino et al for the rs17482078 in their cohort (cases with and without uveitis and discovery and replication data combined, OR = 3.08) [12] but using our sample size (362 patients and 460 controls).

The different association models: genotypic, allelic, dominant and recessive were tested using the χ^2^ test and p values≤0.05 were considered statistically significant. The ORs and 95% confidence intervals (95% CI) were calculated according to Wolf's method using StatCalc program (EPI Info 2002, Centers for Disease Control and Prevention, Atlanta, GA, USA). The linkage disequilibrium solid spine approach was used to define haplotype blocks in the CEU and Spanish populations using the Haploview program (available at the website http://www.broad.mit.edu/mpg/haploview/download.php). The SNP haplotypes frequency estimation in the CEU and Spanish populations was performed using the Haploview sotfware. Conditional logistic regression models were constructed by categorizing all the individuals according to the presence/absence of the HLA-B risk factors (according to a dominant model), the *ERAP1* haplotypes (according to a recessive model) and the *ERAP1* status (yes/no, see results) analyzed with Epi Info 2002.

## Results

The genotyping success ratio was greater than 95% and the study population was found to be in the Hardy–Weinberg equilibrium for all the polymorphisms analyzed (p>0.05). Table S1 in File S1 displays genotype results of the SNPs included in the study and statistical data of the different association models. According to data displayed in Table S2 in File S1, the frequency of the homozygous genotype of the minor allele of the rs17482078 in the healthy Spanish population is 2.6. Taking into account the OR described by Kirino et al in their study (3.08) our a priori statistical power for this SNP was 94.5.

The observed MAFs in the Spanish population were not significantly different to those described in the European population with the exception of the rs30187 which had a significantly higher frequency of the minor allele (T) in our cohort than in the European population included in the 1000 Genome Phase I data project (0.41 vs. 0.34, p = 0.002) but similar to that described in the other Spanish cohort [26]. According to our results and in agreement with a recessive model, frequencies of the homozygous genotypes for the minor alleles of all the SNPs were increased among patients and the ORs ranging from 1.2 to 1.8 although differences were not statistically significant. The original study also reported an epistatic interaction between ERAP1 and HLA-B. Consistent with the original paper, the OR values for the recessive models of the minor alleles of the five SNPs included in the present study were higher in the subgroup of patients with the HLA-B risk factors, although the differences were not statistically significant (Table 1)

**Table 1:** Distribution of the five SNPs studied in *ERAP1* according to a recessive model of the minor allele.

SNP	MA	TEST	Patients	Controls	p	OR
rs27044	G	GG/CC+CG				
		Whole group	46/316	51/409	0.5	1.2
		HLA-B Risk factor group	24/154	7/77	0.2	1.7
rs17482078	T	TT/CC+CT				
		Whole group	17/344	12/446	0.1	1.8
		HLA-B Risk factor group	6/172	1/83	0.3	2.8
rs10050860	T	TT/CC+CT				
		Whole group	17/344	12/446	0.1	1.8
		HLA-B Risk factor group	6/172	1/83	0.3	2.8
rs30187	T	TT/CC+CT				
		Whole group	70/292	73/386	0.2	1.3
		HLA-B Risk factor group	39/137	13/71	0.2	1.5
rs2287987	C	CC/TT+CT				
		Whole group	17/344	12/446	0.1	1.8
		HLA-B Risk factor group	6/172	1/83	0.3	2.8

Individuals with the HLA-B risk factors are subjects bearing HLA-B*51 and/or B*57.

As it was previously described, linkage disequilibrium (LD) among these 5 SNPs was found in the Spanish population. Table 2 shows the four haplotypes (named from H1 to H4) with frequencies greater than 0.05 which were found in our patient and control cohorts. The rs27044G, a risk factor for AS, tags the haplotype H2, whereas the rs17482078T, which has been described to be a risk factor in BD, and also the rs100504860T and rs2287987C tag the haplotype H3. The three haplotypes containing mutations: H2, H3 and H4 were slightly more common among BD patients although the allelic model was not significant. Table 3 displays the conditional multivariate models which include the ERAP1 haplotypes in recessive models and the HLA-B risk factors (HLA-B*51 or B*57 yes/no). The OR values among individuals carrying the HLA-B risk factors were increased more than 2 fold in both, the H2H2 and H3H3 individuals, whereas this value remains the same for H1H1 subjects. Of note, individuals bearing simultaneously H4H4 and the HLA-B risk factors were all patients. In order to analyze the influence of the doses, individuals were grouped in four different categories. Homozygous, subjects with the same set of mutations in all their molecules (“Ho”, H2H2, H3H3, H4H4); double heterozygous, individuals in which all the molecules are mutated but each half has a different set of mutations (“dHe”, H2H3, H2H4 and H3H4); heterozygous, subject having half of molecules with and the other half without mutations (“He”, H1H2, H1H3 and H1H4); and finally, normal individuals, those having no molecules with these mutations (“N”, H1H1). Next, conditional multivariate models taking into account both, the condition HLA-B risk associated with BD (HLA-B*51 or B*57 yes/no) and the *ERAP1* mutation status (Ho, He, dHe and N) were constructed. Table 4 displays the results of this analysis, the OR values among individuals carrying the HLA-B risk factors were increased from 4.51 to 10.72 in the “Ho” positive individuals. Nevertheless, this effect was not observed in “He” nor in “dHe” subjects which have similar results as those obtained in the group “N”.

**Table 2:** *ERAP1* haplotypes obtained by the combination of the five SNPs included in this study.

						Frequency		
Haplotype	rs27044	rs17482078	rs10050860	rs30187	rs2287987	Spanish		CEU
						Patients	Controls	
H1	C	C	C	C	T	0.366	0.401	0.412
H2	**G**	C	C	**T**	T	0.344	0.333	0.244
H3	C	**T**	**T**	C	**C**	0.198	0.186	0.264
H4	C	C	C	**T**	T	0.092	0.079	0.059

Only those haplotypes having frequencies greater than 0.05 are displayed.

Minor alleles are in bold.

**Table 3:** Conditional logistic regression models of HLA-B and of *ERAP1* haplotypes.

HLA-B Risk	H1H1	PatientsN = 343	Controls N = 450	P	OR (95%CI)
+	+	28	13	<10^−5^	4.35 (2.19–8.64)
+	-	150	70	<10^−5^	4.33 (3.07–6.11)
-	+	14	62	0.01	0.46 (0.25–0.84)
-	-	151	305		1.0
HLA-B Risk	H2H2				
+	+	23	6	<10^−5^	8.54 (3.40–21.42)
+	-	155	77	<10^−5^	4.48 (3.20–6.28)
-	+	20	44	>0.05	
-	-	145	323		1.0
HLA-B Risk	H3H3				
+	+	6	1	0.015	13.82 (1.65–115.72)
+	-	172	82	<10^−5^	4.83 (3.49–6.68)
-	+	10	10	>0.05	
-	-	155	357		1.0
HLA-B Risk	H4H4				
+	+	3	0	>0.05	
+	-	175	83	<10^−5^	4.73 (3.44–6.52)
-	+	2	1	>0.05	
-	-	163	366		1.0

**Table 4:** Conditional logistic regression models of HLA-B and three different conditions of *ERAP1*.

Factor B	Ho	Patients N = 343	Controls N = 450	P	OR (95% CI)
+	+	32	7	<10^−5^	10.72 (4.62–24.91)
+	-	146	76	<10^−5^	4.51 (3.20–6.35)
-	+	32	55	>0.05	
-	-	133	312		1.0
Factor B	He				
+	+	79	40	<10^−5^	5.03 (3.16–7.98)
+	-	99	43	<10^−5^	5.86 (3.75–5.14)
-	+	88	171	>0.05	
-	-	77	196		1.0
Factor B	dHe				
+	+	39	23	<10^−5^	3.64 (2.09–6.34)
+	-	139	60	<10^−5^	4.98 (3.45–7.18)
-	+	31	79	>0.05	
-	-	134	288		1.0
Factor B	N				
+	+	28	13	<10^−5^	4.35 (2.19–8.64)
+	-	150	70	<10^−5^	4.33 (3.07–6.11)
-	+	14	62	0.01	0.46 (0.25–0.84)
-	-	151	305		1.0

“Ho” are individuals H2H2,H3H3 and H4H4; “He” are individuals H1H2,H1H3 and H1H4; “dHe” are individuals H2H3,H2H4 and H3H4; “N” are individuals H1H1.

CI confidence interval.

## Discussion

Our results are consistent with the previously described association of *ERAP1* with BD in the Turkish population [12], because the effects detected in our population have the same direction as those described in the original paper, nevertheless, associations and interactions were not statistically significant in the Spanish population.

We decided to attempt the replication in our cohort of BD patients because, additionally to the findings in GWAS, *ERAP1* is an interesting candidate in immune-mediated diseases associated to HLA class I. In fact, the relationship of this gene with AS, which like BD is associated with HLA class I molecules, seems well established. Most of the studies investigating the influence of the *ERAP1* gene in susceptibility to AS include the rs27044 and rs30187 and the authors report the minor alleles of these SNPs as risk factors for this pathology in Caucasian [18], [26]–[30] as well as in Asian populations [31], [32] and in meta-analysis studies [33], [34]. Regarding the other 3 SNPs included in our study, rs17482078, rs100504860 and rs2287987, their minor alleles have been described as protective to AS in some studies but they have been non-associated in others [18], [26]–[28], [31]. Notably, these last 3 SNPs have MAFs lower than rs27044 and rs30187 in different populations and the difference is particularly marked in Asian populations (about 0.07 rs17482078, rs100504860 and rs2287987 vs. 0.50 for rs27044 and rs30187). The low frequency of these SNPs determines a low statistical power, especially in some Asian populations where BD is relatively common, which makes difficult the replication of this finding. In fact, the association between the rs17482078 and susceptibility to this disease which was described in the Turkish population in the original report was not replicated in the same study in the Japanese population. Additionally, the strength of the association could be different among cohorts. Thus, the OR values found in our cohort were lower than those detected in the original study, causing a reduction in statistical power, which *a priori* was higher than 99% for all the SNPs included. Nevertheless, our results were not discrepant with the original report because according to the recessive model found by Kirino et al [12], the frequencies of the homozygous for the minor alleles were higher among patients for all the SNPs and according to an epistatic interaction with the HLA, the OR values were higher among patients with the HLA-B risk factors, although the differences were not significant.

The rs27044 and the rs30187 are in LD with each other (r^2^ = 0.75, D′ = 0.97) and the rs17482078, the rs100504860 and the rs2287987 are also in LD with each other (r^2^ and D′ values greater than 0.90 in the European population). In spite of this strong LD most of the studies in AS include these 5 non-synonimous *ERAP1* polymorphisms. Four major haplotypes (named from H1 to H4 in Table 2) are obtained by the combination of the variations of these five SNPs according to data from CEU and also from our population. The H2 and H3 correspond respectively with risk and protection haplotypes in AS [34], [35], however, our data suggest that both haplotypes could be risk factors in BD. Association of *ERAP1* with AS is restricted to individuals bearing the HLA-B risk factor for this disease, HLA-B27, [35], [36] whereas in psoriasis the relationship between *ERAP1* and HLA-C remains unclear [19], [37]. Althought our results require further confirmation, data displayed in Tables 3 and 4 support a model in which the presence of the same mutation in both chromosomes increased the susceptibility risk in individuals bearing the HLA-B risk factors. *ERAP1* is located in the ER where it is one of the enzymes trimming peptides to reach the optimal size for class I MHC binding. A reduction of the trimming activity for the protective alleles for AS has been found in studies investigating individual changes associated with AS [38]–[40]. Very recently, the influence of the natural haplotypes of *ERAP1* in trimming activity has been explored [41]. These authors suggested that there is a range of trimming activity among the *ERAP1* alleles with some of them encoding molecules that overtrim and others that undertrim. Both conditions, over and under activity could be associated with diseases. The “Ho” status is a scenario in which all the ERAP1 molecules of one individual have the same mutations, producing a lower number of peptides with the appropriate size. Nevertheless, the “dHe” individuals have two different ERAP1 molecules. If one of them over trims whereas the other under trims, the compensation of the enzimatic activity could generate a larger number of units with the appropriate size.

In conclusion, data in the Spanish population were consistent with association between ERAP1 and BD as well as with an epistatic interaction between ERAP1 and HLA-B described in the Turkish population, although our results were not statistically significant.

## Supporting Information

File S1 **Supplementary Tables S1–S2. Table S1:** Different models of association with Behçet disease for the single nucleotide polymorphisms studied in *ERAP1*. **Table S2:** Data of the SNPs studied in *ERAP1* according to a recessive model for the minor alleles.
(DOC)
