# Supplementary material for: Epistatic Interaction of ERAP1 and HLA-B in Behçet Disease: A Replication Study in the Spanish Population
Source: PLoS One. 2014 Jul 14;9(7):e102100. doi: 10.1371/journal.pone.0102100 (PMC4096596; doi:10.1371/journal.pone.0102100)
Supplement: File S1 — Supplementary Tables S1–S2. Table S1: Different models of association with Behçet disease for the single nucleotide polymorphisms studied in ERAP1. Table S2: Data of the SNPs studied in ERAP1 according to a recessive model for the minor alleles. (DOC) [file pone.0102100.s001.doc]

Table S1.

| SNP | MAF | TEST | Patients | Controls | p | OR |
| --- | --- | --- | --- | --- | --- | --- |
| rs27044 | G | Genotypic CC/CG/GG | 154/162/46 | 206/203/51 | 0.7 |  |
|  |  | Allelic C/G | 470/254 | 615/305 | 0.4 | 1.1 |
|  |  | Dominant CC/CG+GG | 154/208 | 206/254 | 0.5 | 1.1 |
|  |  | Recessive CC+CG/GG | 316/46 | 409/51 | 0.5 | 1.2 |
| rs17482078 | T | Genotypic CC/CT/TT | 236/108/17 | 299/147/12 | 0.2 |  |
|  |  | Allelic C/T | 580/142 | 745/171 | 0.6 | 1.1 |
|  |  | Dominant CC/CT+TT | 236/125 | 299/159 | 1.0 | 1.0 |
|  |  | Recessive CC+CT/TT | 344/17 | 446/12 | 0.1 | 1.8 |
| rs10050860 | T | Genotypic CC/CT/TT | 234/110/17 | 298/148/12 | 0.3 |  |
|  |  | Allelic C/T | 578/144 | 744/172 | 0.5 | 1.1 |
|  |  | Dominant CC/CT+TT | 234/127 | 298/160 | 0.9 | 1.0 |
|  |  | Recessive CC+CT/TT | 344/17 | 446/12 | 0.1 | 1.8 |
| rs30187 | T | Genotypic CC/CT/TT | 118/174/70 | 155/231/73 | 0.4 |  |
|  |  | Allelic C/T | 410/314 | 541/377 | 0.3 | 1.1 |
|  |  | Dominant CC/CT+TT | 118/244 | 155/304 | 0.7 | 1.1 |
|  |  | Recessive CC+CT/TT | 292/70 | 386/73 | 0.2 | 1.3 |
| rs2287987 | C | Genotypic TT/CT/CC | 234/110/17 | 298/148/12 | 0.3 |  |
|  |  | Allelic T/C | 578/144 | 744/172 | 0.5 | 1.1 |
|  |  | Dominant TT/CT+CC | 234/127 | 298/160 | 0.9 | 1.0 |
|  |  | Recessive TT+CT/CC | 344/17 | 446/12 | 0.1 | 1.8 |

Table S2.

| SNPs | MA | European MAF | Spanish MAF | Genotypic frequency | | P | OR |
| --- | --- | --- | --- | --- | --- | --- | --- |
|  |  |  |  | Controls | Patients |  |  |
| rs27044 | G | 0.29 | 0.33 | 11.1 | 12.7 | 0.5 | 1.2 |
| rs17482078 | T | 0.22 | 0.19 | 2.6 | 4.7 | 0.1 | 1.8 |
| rs10050860 | T | 0.23 | 0.19 | 2.6 | 4.7 | 0.1 | 1.8 |
| rs30187 | T | 0.34 | 0.41 | 15.9 | 18.8 | 0.2 | 1.3 |
| rs2287987 | C | 0.22 | 0.19 | 2.6 | 4.7 | 0.1 | 1.8 |

SNP Single Nucleotide Polymorphism, MA Minor Allele and MAF Minor Allele Frequency
